# Application of swine manure on agricultural fields contributes to extended-spectrum β-lactamase-producing *Escherichia coli* spread in Tai'an, China

**DOI:** 10.3389/fmicb.2015.00313

**Published:** 2015-04-14

**Authors:** Lili Gao, Jiaqing Hu, Xiaodan Zhang, Liangmeng Wei, Song Li, Zengmin Miao, Tongjie Chai

**Affiliations:** ^1^College of Veterinary Medicine, Shandong Agricultural UniversityTai'an, China; ^2^Sino-German Cooperative Research Centre for Zoonosis of Animal Origin Shandong ProvinceTai'an, China; ^3^Key Laboratory of Animal Biotechnology and Disease Control and Prevention of Shandong ProvinceTai'an, China; ^4^College of Life Sciences, Taishan Medical UniversityTai'an, China

**Keywords:** ESBL-producing *E. coli*, animal manure, ERIC-PCR, agricultural fields, antibiotic resistance gene spread

## Abstract

The prevalence of extended-spectrum beta-lactamase (ESBL)-producing *Escherichia coli* (*E. coli*) is increasing rapidly in both hospital environments and animal farms. A lot of animal manure has been directly applied into arable fields in developing countries. But the impact of ESBL-positive bacteria from animal manure on the agricultural fields is sparse, especially in the rural regions of Tai'an, China. Here, we collected 29, 3, and 10 ESBL-producing *E. coli* from pig manure, compost, and soil samples, respectively. To track ESBL-harboring *E. coli* from agricultural soil, these isolates of different sources were analyzed with regard to antibiotic resistance profiles, ESBL genes, plasmid replicons, and enterobacterial repetitive intergenic consensus (ERIC)-polymerase chain reaction (PCR) typing. The results showed that all the isolates exhibited multi-drug resistant (MDR). CTX-M gene was the predominant ESBL gene in the isolates from pig farm samples (30/32, 93.8%) and soil samples (7/10, 70.0%), but no SHV gene was detected. Twenty-five isolates contained the IncF-type replicon of plasmid, including 18 strains (18/32, 56.3%) from the pig farm and 7 (7/10, 70.0%) from the soil samples. ERIC-PCR demonstrated that 3 isolates from soil had above 90% genetic similarity with strains from pig farm samples. In conclusion, application of animal manure carrying drug-resistant bacteria on agricultural fields is a likely contributor to antibiotic resistance gene spread.

## Introduction

The rapid increase of extended-spectrum beta-lactamases (ESBLs)-producing *Enterobacteriaceae* has attracted worldwide concern. ESBLs are enzymes that make bacteria, especially *Escherichia coli* and *Klebsiella pneumoniae*, resistant to beta-lactam antibiotics including 3rd and 4rd generation cephalosporins to decrease antibiotics available in clinical practices (Bradford, 2001). ESBL-producing *E. coli* were widely found in hospital environments and farms (Edelstein et al., 2003; Smet et al., 2008) and they are able to survive in various natural environments such as feces, soils, and water bodies (Koczura et al., 2012; Blaak et al., 2014; Haque et al., 2014), so ESBL-producing *E. coli* is usually regarded as an indicator bacterium to trace the spread of antibiotic resistance gene (Gao et al., 2014). At present, numerous studies have focused on the occurrence and spread of ESBL genes in hospital environments, waste water treatment plants, water bodies, and food-producing animals (Edelstein et al., 2003; Agerso et al., 2012; Blaak et al., 2014). ESBL-producing *E. coli* have been shown to be able to transmit from animal farms to surrounding environments, even rural water reservoirs (Laube et al., 2014; Von Salviati et al., 2015; Zhang et al., 2015).

In rural areas of China, animal manure has been used to feed agricultural fields as fertilizers in the production of crops, fruits, and vegetables (Huang et al., 2014). But animal feces contain large amounts of antimicrobial-resistant bacteria, including certain pathogenic bacteria such as *Listeria monocytogenes* and *Salmonella* spp. and certain drug-resistant bacteria, such as ESBL-producing *E. coli*, vancomycin-resistant *Enterococcus* and methicillin-resistant *Staphylococcus aureus* (Guber et al., 2007). Animal manure fertilization has been found to increase the abundance of drug-resistant bacteria and the frequency of antibiotic resistance genes in soils (Venglovsky et al., 2009; Marti et al., 2013). Zhu et al. (2013) found diverse and abundant antibiotic resistance genes in compost and soil treated with manure. When animal manure is added into soil in the form of solid or liquid, antibiotic resistance genes can transfer between the same species and even different ones through genetic elements, especially plasmids (D'Costa et al., 2006; Guber et al., 2007; Heuer et al., 2011a). Moreover, antibiotic resistance genes or bacteria in soils could enter into other environments and food chain through various ways to threaten public health. Researchers have found polluted soil could contaminate surface water by over land flow or rainfall (Tate et al., 2000; Curriero et al., 2001). There are also reports about the contamination of resistance gene in vegetables (Ruimy et al., 2010; Reuland et al., 2014). Therefore, investigations on the influence of animal manure application on agriculture are of great significance.

In China, a large number of arable soils have been amended with commercial fertilizers and animal manures (Ju et al., 2007). But, up to date, little information about whether the fertilization model enhances the dissemination of drug-resistant bacteria of animal origins was provided. Previous studies have shown that manure application contributed to the accumulation of resistance genes in soil, mainly sulfonamide resistance genes (Sengeløv et al., 2003; Zhou et al., 2010; Heuer et al., 2011b). In the last decade, the prevalence of ESBL genes has increased rapidly. Meanwhile, ESBL genes that were found to be located on mobile genetic elements, and often associated with other resistance genes, could transfer horizontally between bacteria (Allen, 2014). Thus, this study was performed to assess the dissemination of ESBL-producing *E. coli* of animal origins into agricultural fields.

## Materials and methods

### Sampling sites and collection of samples

Manure samples and soil samples were collected from a pig farm with an intensity of 5000 pigs and its surrounding lands fed with manure between May and July 2014. The farm is located in rural region of Tai'an, China, far away from villages, surrounded by farm land where crops (corn and beans) were planted. Pig manure was piled up and then used as compost into the soil or directly fed the soil by local farmers instead of chemical fertilizer. The soil samples were collected from the cropland which has been receiving compost for at least 3 years.

The pig farm was visited four times. Each time, 10 fecal samples, five composts were collected. At the same time, 20 soil samples were collected from different locations in the surrounding field. Forty soil samples collected from soil treated with chemical fertilizer were used as control. All samples were immediately transported to the lab with an ice box and processed in 6 h.

### Isolation and confirmation of ESBL-producing *E. coli*

About 0.5 g sample soil/feces was dissolved in 5 ml phosphate buffered saline and homogenized. Then 1 ml solution was mixed with 9 ml BHI broth (Haibo, Qingdao, China) for enrichment at 37°C overnight under aerobic conditions. Hundred microliters soil enrichment or 1 loop of the fecal samples enrichment solution was spread onto MacConkey agar (Oxoid, Basingstoke, England) plates with 2 mg/l cefotaxime and then incubated overnight at 37°C.

A presumptive ESBL-producing *E. coli* colony with bright pink or red color was identified by traditional biochemical test including indole, Methyl red-Voges-Proskauer, and citrate biochemical testing and API 20E (bioMerieux, Marcy l'Etoile, France) (Chang et al., 2015).

The screened ESBL-producing *E. coli* isolates were further confirmed by phenotypic confirmatory tests using cefotaxime (30 μg), cefotaxime + clavulanic acid (30 μg/10 μg), ceftazidime (30 μg), ceftazidime + clavulanic acid (30 μg/10 μg) (CLSI, 2013).

### Antimicrobial susceptibility

The confirmed ESBL-producing *E. coli* were subjected to antimicrobial susceptibility tests according to the Clinical and Laboratory Standards Institute (CLSI, 2013) guidelines. Disk diffusion method was used to determine resistance profile of the isolates on Mueller-Hinton agar (Haibo, Qingdao, China). Eight drug classes containing 14 antibiotics (Oxoid, England) were included representing the most frequently used antibiotics in animals. Penicillins were represented by amoxicillin (AML). Cephalosporins included ceftiofur (CET), cephalothin (KF), cefotaxime (CTX), and ceftriaxone (CRO). Carbopenems were represented by imipenem (IPM). Aminoglycosides were represented by gentamicin (GM), kanamycin (K), and amikacin (AK). Quinolones included nalidixic acid (NA) and ciprofloxacin (CIP). Tetracyclines were represented by tetracycline (TE). Chloramphenicol (C) and florfenicol (FFC) were also included. The standard *E. coli* ATCC 25922 was used as quality control strain (Zhang et al., 2014). *E. coli* isolates resistant to three or more categories were regarded as multi-drug resistant (MDR), resistant to one or two categories regarded as extensively drug-resistant (XDR) and resistant to all antimicrobial categories considered as pandrug-resistant (PDR) (Magiorakos et al., 2012).

### Detection of ESBL genes

Major β-lactamase genes detected in food-producing animals in China, *bla*_TEM_, *bla*_SHV_, *bla*_CTX−M_, *bla*_CMY−2_, and *bla*_OXA_, were amplified by PCR (Zhao et al., 2001; Monstein et al., 2007; Shaheen et al., 2011). Groups of CTX-M-positive *E. coli* were further determined using specific group primers for CTX-M-1, CTX-M-2, and CTX-M-9 (Batchelor et al., 2005). The PCR products were purified using a PCR purification kit (TianGen, Beijing, China) and then sequenced bi-directionally with the same primers by ABI 3730 DNA sequencer (Applied Biosystems, Foster City, CA, USA) (Zhang et al., 2014). All gene sequences were subjected to BLASTn analysis in GenBank (http://www.ncbi.nlm.nih.gov/) or β-lactamase classification system (http://www.lahey.org/studies/webt.asp) to confirm subtypes of ESBL-encoding genes (Zheng et al., 2012).

### Plasmid replicon typing

The plasmid replicon types of the ESBL-producing *E. coli* were detected using the inc/rep PCR method (a PCR method based on replicons of major plasmid incompatibility groups) (Carattoli et al., 2005). Multiplex- and simplex-PCR were used to recognize 18 plasmid incompatibility groups among *Enterobacteriaceae* including FIA, FIB, FIC, HI1, HI2, I1-Ig, L/M, N, P, W, T, A/C, K, B/O, X, Y, F, and FIIA and performed as previously described (Carattoli et al., 2005).

### ERIC-PCR analysis

Twenty-one ESBL-producing *E. coli* isolates from pig farm samples and seven isolates from treated soil samples were selected randomly and subjected to enterobacterial repetitive intergenic consensus sequence (ERIC-PCR) to analyze the similarity of the strains from manure, compost and treated soil. The PCR procedure and conditions were performed as previously described (Koczura et al., 2012). The amplified products were separated in 2% agarose gel by electrophoresis. The presence or absence of the amplified bands on the gel were recorded as 1 or 0 for further analysis. NTSYS-pc (Version 2.10) was used to do cluster analysis based on Dice's similarity coefficient with a 1% position tolerance and the unweighted pair group method using arithmetic averages (UPGMA). Isolates of different origins or the same origin with above 90% similarity were treated as the same strain (Edelstein et al., 2003; Yuan et al., 2010).

## Results

### Isolation of ESBL-producing *E. coli*

A total of 42 ESBL-producing *E. coli* were isolated from the 180 samples. There were 32 isolates from pig farm samples, including 29 from manure and three from compost samples. From the soil samples treated with compost or manure, 10 ESBL-producing *E. coli* were isolated. However, no ESBL-producing isolate was obtained from soil treated with chemical fertilizer (Table 1).

**Table 1:** **Isolation of ESBL-producing *E. coli* from manure, compost, treated soil, and untreated soil**.

**Origin**	**No. of samples**	**No. of ESBL-producer**	**Isolation rate (%)**
Manure	40	29	72.5
Compost	20	3	15.0
Treated soil	80	10	12.5
Untreated soil	40	0	0
Total	180	42	23.3

### Antimicrobial susceptibility

All the 42 ESBL-producing *E. coli* from manure, compost, and treated soil showed multiple drug resistance to antibiotics tested and the isolates from different origins had similar drug-resistant spectrum against the 14 antibiotics. Forty-two isolates were all 100% resistant to AML and KF, but susceptible to IMP. Additionally, isolates from different sources had above 60% resistance to CET, K, TE, C, and FFC (Table 2 and Table S1).

**Table 2:**
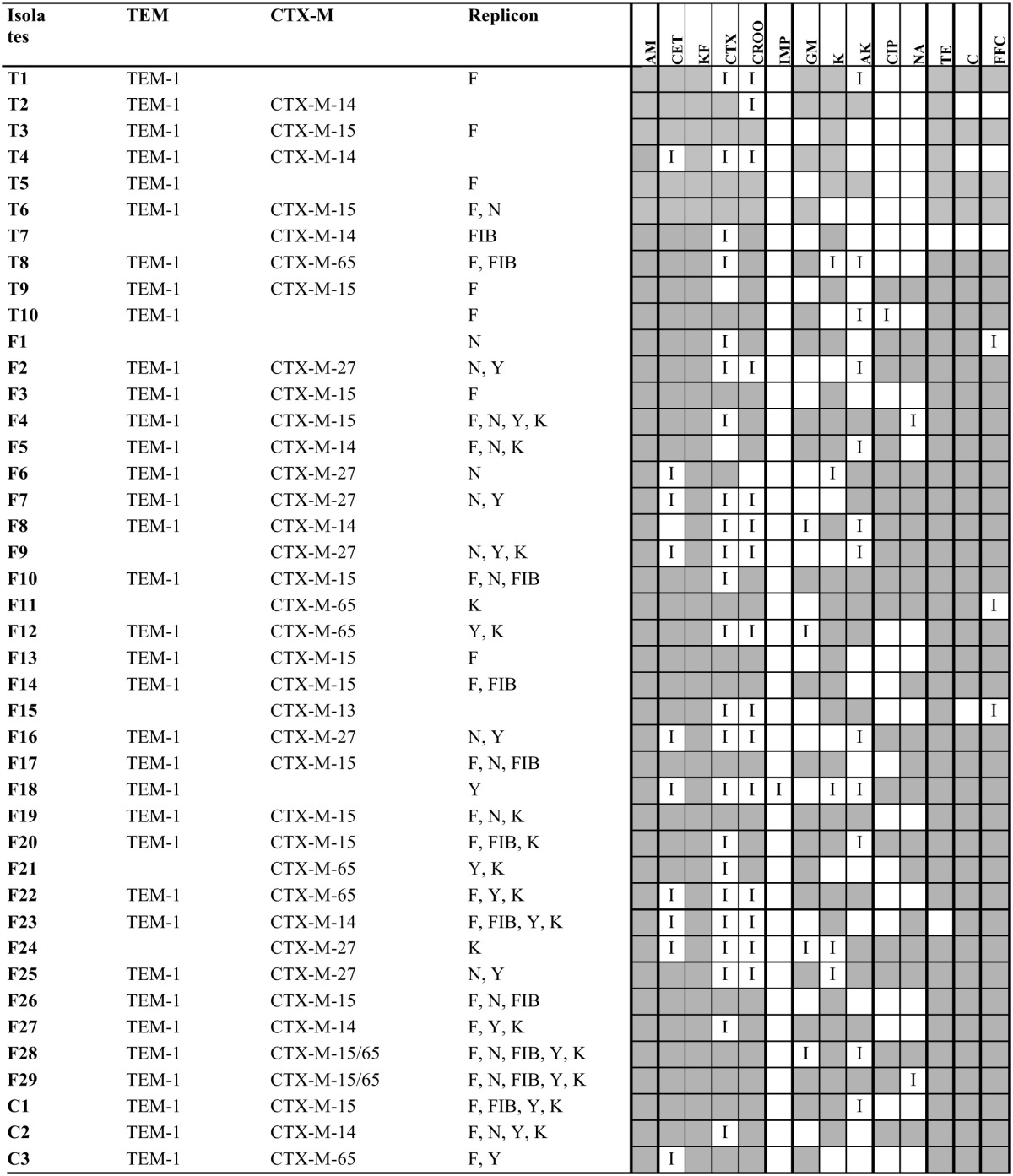
**Characteristics of the 42 ESBL-producing *E. coli* from different samples including β-lactamase genes, replicon type, and resistance profile**.

*T, ESBL-producing E. coli isolates from soil; F, ESBL-producing E. coli from feces; C, ESBL-producing E. coli from compost*.

*The gray color represents resistant, the white color means susceptible and “I” represents intermediate. Different classifications of antibiotics were distinguished by bold lines. CET, ceftiofur; AML, amoxicillin; KF, cephalothin; CTX, cefotaxime; IPM, imipenem; CRO, ceftriaxone; GM, gentamicin; K, kanamycin; AK, amikacin; NA, nalidixic acid; CIP, ciprofloxacin; TE, tetracycline; C, chloramphenicol; FFC, florfenicol*.

### Characterization of β-lactamase genes

Among the 42 ESBL-producing *E. coli*, 37 isolates carried *bla*_CTX−M_(37/42, 86.0%), including 30 isolates from the pig farm (30/32, 93.8%), and seven from treated soil (7/10, 70.0%). Thirty-one isolates carried *bla*_CTX−M+TEM_ gene, including 25 isolates (25/32, 78.1%) from the pig farm and six (6/10, 60.0%) from treated soil. The other three genes (SHV, CMY-2 and OXA) were all not detected.

Sequence and blast results showed that the most prevalent ESBL gene in pig farm samples was *bla*_CTX−M−15_, which was found in 12 isolates, followed by *bla*_CTX−M−27_ and *bla*_CTX−M−65_ respectively detected in seven isolates. Five isolates from the pig farm carried *bla*_CTX−M−14_ and one isolate harbored *bla*_CTX−M−13_. Two isolates from fecal samples carried both *bla*_CTX−M−15_ and *bla*_CTX−M−65_ gene. Both *bla*_CTX−M−15_ and *bla*_CTX−M−14_ genes were the common ESBL genes in isolates from soil samples and respectively detected in three strains as shown in Table 2.

Five isolates of the 42 ESBL-producing *E. coli* did not carry ESBL genes (three from soil samples and two from pig farm samples). Three isolates from soil samples and one from manure only carried *bla*_TEM−1_ gene. No β-lactamase gene was found in one isolate from manure.

### Plasmid replicon type

Among the 42 ESBL-producing *E. coli* of different origins, IncF was the most prevalent replicon type in the isolates from the pig farm (18/32, 56.3%) and treated soil samples (7/10, 70%). IncN was detected in 16 ESBL-producers from pig farm samples and one from soil samples. IncFIB was found in nine isolates from pig farm samples and two from soil samples. Four isolates were not typed including both two from pig farm samples and soil samples as shown in Table 2.

Isolates from pig farm samples showed a higher diversity of inc/rep than that from soil samples. IncK and Y were detected in 16 and 17 isolates from pig farm samples respectively, while none of them was found in soil samples.

### Similarity analysis of ESBL-producing *E. coli* from manure, compost, and treated soil

**Figure 1:**
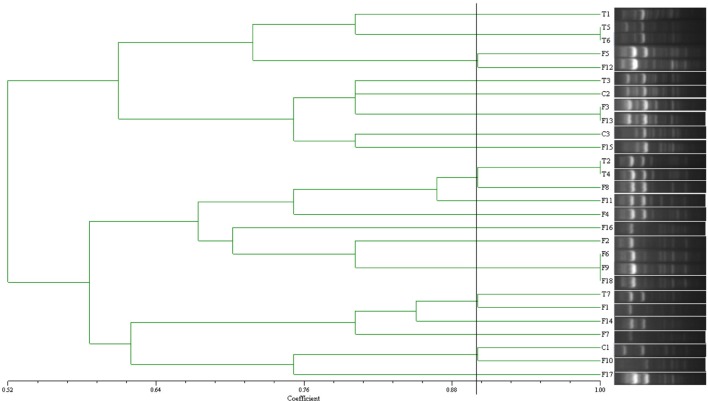
**Dendrogram of ERIC-PCR results of 28 ESBL-producing *E. coli* originating from manure (F), compost (C), and soil (T)**.

The similarity between ESBL-producing *E. coli* from pig farm and treated soil was between 70 and 100%. Three isolates (T2, T4, and T7) from treated soil had above 90% similarity with those from pig manure and compost samples (F8 and F1) (Figure 1), which suggested that they might come from the same strain.

## Discussion

Animal wastes without composting process have been widely used in agricultural fields, especially in developing countries to improve the soil fertility and structure (Kumar et al., 2005; Hu et al., 2010). However, animal original bacteria carrying antibiotic resistance genes can regrow to high levels under favorable condition (Ghosh and LaPara, 2007; Marti et al., 2013; Chen and Jiang, 2014). Wide application of animal manure to agricultural fields has raised a concern about antibiotic resistance genes spread. Once antimicrobial-resistant bacteria from animal feces enter into arable soils, mobile genetic elements can transmit between the same or different species of bacteria under some conditions, especially between *Enterobacteriaceae* (Rensing et al., 2002; Heuer and Smalla, 2007). Additionally, these bacteria can spread to other environments through rainwater or other routes (Khaleel et al., 1982; Curriero et al., 2001).

Soil harbored a vast diversity of microorganisms, which was considered as a potential reservoir for antibiotic resistance (Forsberg et al., 2012). When drug-resistant bacteria of animal origins enter into this community, they could exchange resistance genes with soil-dwelling organisms. In this study, we isolated ESBL-producing *E. coli* from one pig farm as an indicator to better understand the pollution of ESBL-producing *E. coli* from animal manure on agricultural fields. We found that ESBL producers from manure, compost, and soil showed a remarkable similarity in terms of resistance phenotypes, ESBL genes, plasmid replicon type, and genomic characterization.

Previous studies have demonstrated that animal feces carrying drug-resistant bacteria can lead to antibiotic resistance genes spread, which attracted wide concern worldwide (Heuer et al., 2011a). In China, a large of arable soils have been amended with commercial fertilizers and animal manure (Huang et al., 2014), therefore it is of importance to investigate whether this fertilization model can enhance the dissemination of drug-resistant bacteria of animal origins can disseminate into the surrounding soil. In this study, we isolated ESBL-producing *E. coli* from pig farm samples (manure and compost) and soil treated with pig manure, but no isolate was detected in control soil samples only treated with chemical fertilizer. These results indicated that pig manure may be a likely contributor of drug-resistant bacteria or genes, and antibiotic selection may be another reason. Additionally, the presence of ESBL-producers in soils may be related with the sampling time and the frequency of fertilization (Jensen et al., 2001; Sengeløv et al., 2003; Riber et al., 2014), which is required to be further studied.

The 42 ESBL-producing *E. coli* all showed multi-drug resistance to 14 antibiotics commonly used in the local clinics, and the isolates both from pig farm samples and treated soil had the similar characteristics of resistance to a large extend. The close relatedness also indicated that application of pig manure to agricultural fields may play an important role in the dissemination of drug-resistant bacteria or genes of animal origins. ESBL-producers from different sources showed high resistance to the β-lactam antibiotics. It may be related with the use of ceftiofur that was allowed to be used in veterinary medicine in China (Guo et al., 2014).

CTX-M gene was the predominant ESBL genes in pig farm samples, which was also the main ESBL gene detected in soil samples as previous studies (Pitout and Laupland, 2008; Ewers et al., 2012). CTX-M-15, CTX-M-14, and CTX-M-65 genes were found in ESBL-producing *E. coli* of different origins. CTX-M-15 was detected in 12 isolates in pig farms accounting for 37.5%, similar prevalence 30% was observed in treated soil samples. IncF was the dominant replicon type in samples from pig farms and soil samples detected in 18 isolates from pig farm samples and seven from soil. Additionally, ERIC-PCR of the ESBL-producing *E. coli* showed four isolates from treated soil exhibited above 90% similarity with fecal isolates. The similar characteristics suggested that isolates in soil may mainly come from the manure or compost. The result showed that pig manure may be a likely contributor of drug-resistant bacteria or genes, including ESBL producers. However, isolates from the pig farm showed a high diversity of replicon or ESBL genes, which may be due to the different evolution and selective pressure of those isolates.

The bacteria community of arable soil was closely related with organic fertilizer applications. Previous studies have demonstrated that drug-resistant genes were found in the arable soil amended with manures (Sengeløv et al., 2003; Heuer et al., 2011a) and the repeated application of manure could increase the abundance of sulfonamide resistance gene (Heuer et al., 2011b). ESBL-producing *E. coli* have been detected on the surface of the ground in the vicinity of the animal farms (Walsh and Duffy, 2013; Laube et al., 2014).

In summary, ESBL-producing isolates from compost, treated soil, and manure showed high overlaps in terms of resistance phenotypes, ESBL genes, plasmid replicon type, and genomic backbone characterization, which implies the dissemination of resistance bacteria or genes of animal origins to soil that treated with animal manure.

### Conflict of interest statement

The authors declare that the research was conducted in the absence of any commercial or financial relationships that could be construed as a potential conflict of interest.
