# Supplementary material for: Application of swine manure on agricultural fields contributes to extended-spectrum β-lactamase-producing Escherichia coli spread in Tai'an, China
Source: Front Microbiol. 2015 Apr 14;6:313. doi: 10.3389/fmicb.2015.00313 (PMC4396445; doi:10.3389/fmicb.2015.00313)
Supplement: Supplementary file 1 [file Table1.DOCX]

**Table S1 The diameter of the inhibition zone about the 14 antibiotics (mm).**

Different classifications of antibiotics were distinguished by bold lines.

CET, ceftiofur; AML, amoxicillin; KF, cephalothin; CTX, cefotaxime; IPM, imipenem; CRO, ceftriaxone; GM, gentamicin; K, kanamycin; AK, amikacin; NA, nalidixic acid; CIP, ciprofloxacin; TE, tetracycline; C, chloramphenicol; FFC, florfenicol.

| **Strains** | **AML** | **EFT** | **KF** | **CTX** | **CRO** | **IPM** | **GM** | **K** | **AK** | **CIP** | **NA** | **TE** | **C** | **FFC** |
| --- | --- | --- | --- | --- | --- | --- | --- | --- | --- | --- | --- | --- | --- | --- |
| **T1** | 0 | 12 | 0 | 23 | 20 | 32 | 0 | 0 | 16 | 30 | 25 | 0 | 0 | 0 |
| **T2** | 0 | 12 | 0 | 0 | 21 | 30 | 0 | 9 | 13 | 30 | 20 | 0 | 20 | 21 |
| **T3** | 0 | 0 | 0 | 15 | 17 | 30 | 18 | 0 | 18 | 28 | 24 | 0 | 0 | 0 |
| **T4** | 0 | 20 | 0 | 24 | 20 | 28 | 0 | 10 | 18 | 32 | 23 | 0 | 22 | 22 |
| **T5** | 0 | 9 | 0 | 13 | 11 | 30 | 18 | 0 | 12 | 30 | 22 | 0 | 0 | 9 |
| **T6** | 0 | 0 | 0 | 12 | 11 | 32 | 0 | 18 | 20 | 22 | 21 | 0 | 0 | 0 |
| **T7** | 0 | 13 | 0 | 23 | 12 | 30 | 18 | 11 | 20 | 26 | 22 | 21 | 20 | 22 |
| **T8** | 0 | 13 | 0 | 23 | 12 | 30 | 11 | 16 | 15 | 27 | 21 | 9 | 0 | 9 |
| **T9** | 0 | 10 | 0 | 26 | 12 | 24 | 20 | 0 | 17 | 12 | 0 | 0 | 0 | 0 |
| **T10** | 0 | 10 | 0 | 12 | 10 | 30 | 0 | 17 | 15 | 18 | 20 | 0 | 0 | 0 |
| **F1** | 0 | 12 | 0 | 23 | 11 | 29 | 11 | 0 | 18 | 0 | 0 | 0 | 0 | 15 |
| **F2** | 0 | 13 | 0 | 24 | 22 | 26 | 17 | 20 | 16 | 13 | 0 | 0 | 0 | 0 |
| **F3** | 0 | 10 | 0 | 17 | 11 | 27 | 18 | 0 | 17 | 25 | 21 | 0 | 0 | 0 |
| **F4** | 0 | 0 | 0 | 23 | 18 | 28 | 12 | 0 | 12 | 0 | 18 | 0 | 0 | 0 |
| **F5** | 0 | 0 | 0 | 26 | 10 | 25 | 0 | 0 | 15 | 14 | 23 | 11 | 0 | 10 |
| **F6** | 0 | 18 | 0 | 13 | 25 | 28 | 16 | 16 | 14 | 13 | 0 | 0 | 11 | 0 |
| **F7** | 0 | 19 | 0 | 25 | 20 | 27 | 18 | 18 | 14 | 14 | 0 | 0 | 0 | 0 |
| **F8** | 0 | 21 | 0 | 25 | 20 | 25 | 14 | 11 | 16 | 10 | 0 | 0 | 0 | 9 |
| **F9** | 0 | 20 | 0 | 24 | 21 | 26 | 15 | 18 | 16 | 12 | 0 | 0 | 0 | 9 |
| **F10** | 0 | 12 | 0 | 24 | 16 | 30 | 0 | 0 | 0 | 10 | 0 | 0 | 0 | 0 |
| **F11** | 0 | 0 | 0 | 15 | 15 | 30 | 15 | 12 | 14 | 12 | 0 | 10 | 12 | 17 |
| **F12** | 0 | 13 | 0 | 23 | 22 | 30 | 13 | 0 | 0 | 25 | 20 | 0 | 0 | 0 |
| **F13** | 0 | 10 | 0 | 13 | 11 | 28 | 17 | 0 | 21 | 30 | 22 | 0 | 0 | 0 |
| **F14** | 0 | 11 | 0 | 13 | 13 | 30 | 0 | 0 | 20 | 22 | 0 | 10 | 0 | 0 |
| **F15** | 0 | 13 | 0 | 23 | 20 | 30 | 17 | 0 | 12 | 27 | 24 | 0 | 24 | 16 |
| **F16** | 0 | 18 | 0 | 24 | 20 | 30 | 15 | 18 | 15 | 12 | 0 | 0 | 0 | 0 |
| **F17** | 0 | 10 | 0 | 14 | 13 | 31 | 0 | 0 | 18 | 22 | 0 | 0 | 0 | 0 |
| **F18** | 0 | 19 | 0 | 23 | 21 | 20 | 16 | 17 | 15 | 12 | 0 | 0 | 0 | 0 |
| **F19** | 0 | 10 | 0 | 11 | 10 | 30 | 0 | 0 | 14 | 30 | 22 | 11 | 0 | 0 |
| **F20** | 0 | 11 | 0 | 23 | 12 | 29 | 0 | 0 | 16 | 11 | 0 | 0 | 0 | 0 |
| **F21** | 0 | 10 | 0 | 24 | 11 | 30 | 0 | 18 | 17 | 21 | 0 | 0 | 0 | 0 |
| **F22** | 0 | 18 | 0 | 23 | 21 | 26 | 0 | 0 | 0 | 25 | 20 | 0 | 10 | 0 |
| **F23** | 0 | 20 | 0 | 25 | 22 | 28 | 21 | 0 | 18 | 23 | 11 | 0 | 0 | 0 |
| **F24** | 0 | 18 | 0 | 24 | 20 | 27 | 14 | 14 | 13 | 14 | 0 | 22 | 0 | 0 |
| **F25** | 0 | 12 | 0 | 23 | 20 | 25 | 12 | 14 | 14 | 13 | 0 | 0 | 0 | 9 |
| **F26** | 0 | 12 | 0 | 17 | 13 | 30 | 17 | 0 | 20 | 30 | 22 | 0 | 0 | 0 |
| **F27** | 0 | 11 | 0 | 23 | 13 | 31 | 0 | 0 | 0 | 28 | 20 | 0 | 0 | 0 |
| **F28** | 0 | 0 | 0 | 11 | 10 | 30 | 14 | 0 | 15 | 0 | 0 | 0 | 0 | 0 |
| **F29** | 0 | 0 | 0 | 10 | 10 | 26 | 12 | 0 | 14 | 0 | 15 | 0 | 0 | 0 |
| **C1** | 0 | 10 | 0 | 13 | 12 | 26 | 11 | 10 | 16 | 22 | 20 | 0 | 0 | 0 |
| **C2** | 0 | 0 | 0 | 23 | 18 | 32 | 15 | 0 | 18 | 0 | 10 | 0 | 0 | 0 |
| **C3** | 0 | 18 | 0 | 18 | 11 | 28 | 11 | 18 | 20 | 21 | 23 | 0 | 0 | 0 |
